# Safety and effectiveness of a novel electric femoral artery compression device for hemostasis after femoral artery puncture

**DOI:** 10.3389/fneur.2025.1582310

**Published:** 2025-05-19

**Authors:** Zhihong Zhong, Hongyang Ni, Xiao Chen, Jun Zhu, Hong Jiang, Jinqing Hu, Dong Lin, Liuguan Bian

**Affiliations:** Department of Neurosurgery, Ruijin Hospital, Shanghai Jiao Tong University School of Medicine, Shanghai, China

**Keywords:** femoral arterial puncture, percutaneous endovascular procedure, hemostasis, compression device, percutaneous intervention

## Abstract

**Objective:**

This study aimed to investigate the hemostatic efficacy of a novel electric femoral artery compression device in patients who underwent percutaneous endovascular procedure through femoral artery puncture.

**Methods:**

We retrospectively reviewed 1,086 patients treated with the electric compression device (ECD) and/or vascular closure device (VCD) for hemostasis of femoral catheterization sites. The clinical success of hemostasis and complications of femoral artery puncture site were collected and analyzed.

**Results:**

A total clinical success rate of 84.8% was achieved. Minor complications occurred in 161 of 1,086 patients (14.8%), and major complications occurred in 4 of 1,086 patients (0.4%). Combined application of ECD and VCD showed a lower rate of complications than using ECD or VCD alone in patients who underwent treatment of antiplatelet agents and anticoagulant.

**Conclusion:**

ECD is a safe and effective tool for hemostasis in femoral artery puncture site in percutaneous endovascular procedure with high clinical success and low complication rates.

## Introduction

Recently, percutaneous endovascular procedure is increasingly performed for angiography and interventional therapy as its less invasiveness. The common femoral artery is the common puncture site for arterial access. Hemostasis at the puncture site is an important step after percutaneous endovascular procedure. Improper compression of the puncture site may increase the probability of vascular complications (1).

Manual compression is the most used but time-consuming and laborious hemostasis method after femoral artery puncture. Also, it requires prolonged bed rest and increases the level of patient discomfort (2). Several alternative methods for hemostasis at the femoral artery puncture site have been applied, including vascular closure devices (VCDs), such as Starclose, ProGlide, and AngioSeal, and other mechanical compression devices, such as God’s Hand pneumatic compression device and butterfly compression device (3, 4). However, VCDs may carry the risk of complications, including arterial stenosis or occlusion due to local suturing, arterial embolism due to embolic objects into the blood vessel, and local infection (5–8). In addition, the efficacy of God’s Hand device and butterfly compression device in patients who underwent puncture with arterial sheath > 6 Fr is still unclear (3, 4).

In this study, we introduced a novel electric femoral artery compression device which might be safe and comfortable for patients and time-saving and labor-saving for intervention physician. The purpose of the present study was to examine the feasibility and safety of the electric compression device (ECD) for hemostasis in percutaneous endovascular procedure by femoral arterial access.

## Materials and methods

### Patients

This study included 1,086 consecutive patients in whom the hemostasis of femoral catheterization sites was achieved using ECD and/or VCD between January 2021 and December 2022. The inclusion criteria for application of ECD or VCD were no history of coagulation dysfunction, no severe peripheral vascular disease, and an arterial sheath ≤ 8 Fr. Patients who were marked obesity, underwent endovascular procedure via radial approach, or declined to use the devices were excluded. Patients were divided into groups A, B, and C according to the method of hemostasis of the puncture site: (1) group A, hemostasis of femoral puncture site was achieved using VCD; (2) group B, ECD was used to achieve hemostasis, and (3) group C, hemostasis effect was achieved by the combined application of the ECD and VCD. Each group was further divided into two subgroups depending on whether antiplatelet agents and anticoagulant were used or not. In subgroups A1, B1, and C1, the patients underwent percutaneous endovascular procedure without treatment of antiplatelet agents or anticoagulant. In subgroups A2, B2, and C2, the patients were treated with dual antiplatelet agents perioperatively and anticoagulant during the endovascular procedure. The patient characteristics and interventional procedural details are summarized in Table 1. The study was approved by the medical ethics committee of Ruijin Hospital affiliated to Shanghai Jiao Tong University School of Medicine.

**Table 1:** Baseline characteristics of patients.

Characteristics	Group A (*N* = 507)	Group B (*N* = 294)	Group C (*N* = 285)	*p* value
Age, X ± SD, y	61 ± 12	63 ± 13	60 ± 12	0.041 (B vs. C)
Sex, male, *N* (%)	320 (63.1)	205 (69.7)	165 (57.9)	0.012 (B vs. C)
BMI, X ± SD	24.73 ± 3.10	24.29 ± 3.26	24.76 ± 2.8	0.100
Smoking, *N* (%)	136 (26.8)	123 (41.8)	93 (32.6)	<0.001(A vs. B)
Hypertension, *N* (%)	389 (76.7)	226 (76.9)	194 (68.1)	0.017 (A vs. C)
Diabetes mellitus, *N* (%)	160 (31.6)	117 (39.8)	84 (29.5)	0.017 (B vs. C)
Heart disease, *N* (%)	105 (20.7)	50 (17.0)	40 (14.0)	0.056
Renal dysfunction, *N* (%)	31 (6.1)	18 (6.1)	13 (4.6)	0.623
INR, X ± SD	0.97 ± 0.07	0.96 ± 0.07	0.96 ± 0.07	0.003 (A vs. B)
Platelets count, X ± SD	211 ± 56	211 ± 55	212 ± 61	0.993
Antiplatelet, *N* (%)				0.950
None	101 (19.9)	59 (20.1)	51 (17.9)	
Single	47 (9.3)	27 (9.2)	25 (8.8)	
Dual	359 (70.8)	208 (70.7)	209 (73.3)	
Procedure type, *N* (%)				<0.001 (A vs. B; A vs. C; B vs. C)
Angiography	171 (33.7)	118 (40.1)	59 (20.7)	
Stenting	214 (42.2)	148 (50.3)	120 (42.1)	
Embolization	122 (24.1)	28 (9.5)	106 (37.2)	
Sheath size, *N* (%)				<0.001 (A vs. B; A vs. C; B vs. C)
5F	175 (34.5)	118 (40.1)	59 (20.7)	
6F	71 (14.0)	9 (3.1)	72 (25.3)	
8F	261 (51.5)	167 (56.8)	154 (54.0)	
Duration of catheterization, X ± SD	1.22 ± 0.66	1.06 ± 0.62	1.51 ± 0.73	<0.001 (A vs. B; A vs. C; B vs. C)

BMI, body mass index; INR, international normalized ratio; SD, standard deviation.

### Device and hemostasis procedure

In group A, The ProGlide VCD (Abbot Vascular, USA) was applied. ProGlide was a 6 Fr suture mediated closure system designed for closure of femoral artery puncture sites up to 8 Fr. Briefly, after removal of the arterial sheath, the closure device was inserted over a wire, and then the preformed suture loop was advanced and tightened to obtain hemostasis (9).

**Figure 1:**
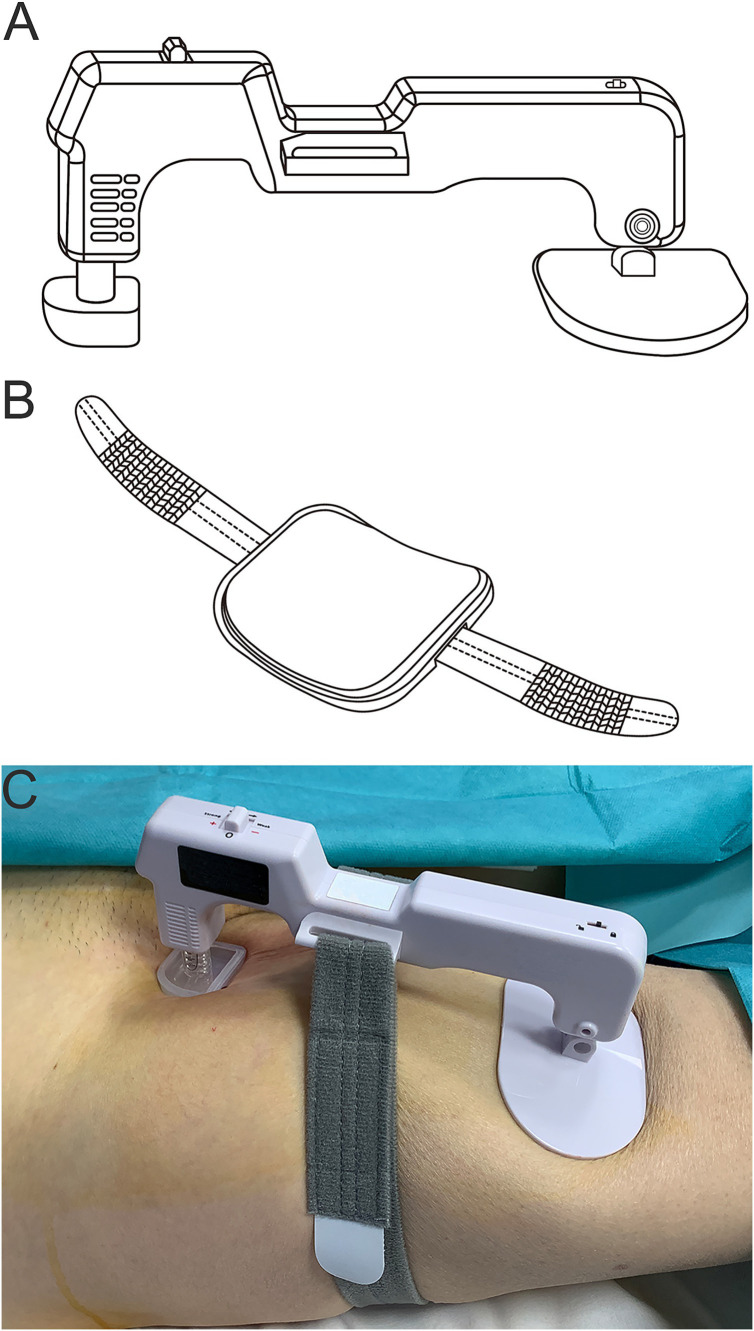
Schematic diagram of the electric compression device. **(A)** The “C-shaped” main body. **(B)** The supplementary pad with the supplementary belt. **(C)** Hemostasis of the femoral artery puncture.

In group B, the Efinger ECD was used. It was a single-use, disposable ECD for hemostasis of the femoral puncture site. It consisted of a “C-shaped” main body and a supplementary pad with a supplementary belt. The main body had an adjustable tip on one side of the “C-shape” and a pad on another side. In brief, hemostasis was achieved as follows: With the sheath in the femoral artery, the supplementary pad with the supplementary belt was put under the upper third of the thigh of the patient first. Next, the main body was secured over the puncture site with the center of the adjustable tip positioning 1 cm proximal to the sheath entry site. The supplementary belt was connected to the main body to reinforce the attachment of the main body to the skin. Then the sheath was gradually removed, meanwhile the adjustable tip was adjusted until no signs of bleeding were present. In case of any signs of ischemia of distal limb during application of ECD, the adjustable tip would be timely adjusted until the ischemic signs disappeared. (Figure 1).

In group C, the combined application of the ProGlide VCD and Efinger ECD was performed. After removal of the arterial sheath, VCD was used first and then ECD was secured over the puncture site.

After successful hemostasis, the patient was transferred to the ward. The Efinger ECD was kept at the femoral puncture site in groups B and C. Then the bleeding status, ischemic signs and any other complications were assessed every 30 min for 2 h and every 60 min after 2 h. The Efinger ECD was removed after 4 h of application. The puncture site was observed continually to record the complications, including bleeding, hematoma, subcutaneous bruising, pseudoaneurysm or arteriovenous fistula until ambulation.

### Assessment

Clinical information including baseline clinical characteristics, endovascular procedural details, outcomes and complications of hemostasis were reviewed. The baseline clinical characteristics included patients’ age, sex, body mass index, smoking history, and a history of hypertension, diabetes mellitus, heart disease, and chronic kidney disease. Endovascular procedural details included the procedure type, sheath size, and time with sheath *in situ*. In addition, the data of pre-operative and post-operative hemoglobin level was collected to evaluate blood loss after endovascular procedure. The clinical success of hemostasis was defined as the ability of ambulation of the patient after four hours of bed rest without any complications related to hemostasis.

The complications were classified according to the Society of Interventional Radiology guidelines (10). Major complications were defined as those that involved additional interventional procedures, prolonged hospitalization, permanent adverse sequelae, or death. All other complications were classified as minor, including mild bleeding and mild hematoma, which needed no additional therapy, had no consequence, and was managed by observation only (10). Moderate and severe subcutaneous bruising at the puncture site which was attributed to oozing of femoral artery was also assessed. Moderate subcutaneous bruising was defined as the area more than 5*5 cm but less than 15*15 cm, while the area which was more than 15*15 cm was considered as severe oozing. In this study, mild subcutaneous bruising in which the area was less than 5*5 cm was not calculated in minor complication.

### Statistical analysis

All continuous data were presented as mean ± standard deviation (SD) and compared using the analysis of variance (ANOVA). Categorical data were presented as N (%) and compared using the chi-square test. Logistic regression analysis was conducted to estimate the odds ratio (OR) of puncture complications (including minor and major complications) and its 95% confidence interval (95% CI) with compression methods adjusting for age, sex, BMI, smoking, hypertension, diabetes, renal dysfunction, platelets count, INR, duration of catheterization, and sheath size stratified by whether using antiplatelet. Statistical analyses were performed with SPSS Statistics (version 20.0, SPSS Inc., Chicago, IL, United States).

## Results

A total clinical success rate was 84.8% (921/1086) in this study. The clinical success rate was 83.6% (424/507) in the Group A, 82.7% (243/294) in the Group B, and 89.1% (254/285) in the Group C, respectively. In the patients without treatment of antiplatelet agents or anticoagulant, clinical success was achieved in 88 of 101 (87.1%) in the subgroup A1, 51 of 59 (86.4%) in the subgroup B1, and 45 of 51 (88.2%) in the subgroup C1. In the patients who underwent treatment of antiplatelet agents and/or anticoagulant, clinical success was achieved in 336 of 406 (82.8%) in the subgroup A2, 192 of 235 (81.7%) in the subgroup B2, and 209 of 234 (89.3%) in the subgroup C2. The subgroup C2 showed a higher clinical success rate than the other two subgroups (A2 and B2).

A hemoglobin decrease of more than 10 g/L was observed in 526 of 1,086 patients (48.4%): 251 of 507 (49.5%) in the group A, 143 of 294 (48.6%) in the group B, and 132 of 285 (46.3%) in the group *C. minor* complications occurred in 161 of 1,086 patients (14.8%): 81 of 507 (16.0%) in the group A, 50 of 294 (17.0%) in the group B, and 30 of 285 (10.5%) in the group *C. major* complications occurred in 4 of 1,086 patients (0.4%): two in group A, one in group B, and one in group C (Table 2).

**Table 2:** Clinical outcome of each group of patients.

Clinical outcome	Group A (*N* = 507)	Group B (*N* = 294)	Group C (*N* = 285)	*p* value
Clinical success, *N* (%)	424 (83.6)	243 (82.7)	254 (89.1)	0.055
Time to hospital discharge, X ± SD, d	4 ± 10	3 ± 5	4 ± 6	0.494
Hemoglobin decrease > 10 g/L, *N* (%)	251 (49.5)	143 (48.6)	132 (46.3)	0.512
10–20 g/L, *N* (%)	203 (40.0)	118 (40.1)	115 (40.4)	
>20 g/L, *N* (%)	48 (9.5)	25 (8.5)	17 (6.0)	
Minor complication, *N* (%)	81 (16.0)	50 (17.0)	30 (10.5)	0.055
Hematoma, *N* (%)	9 (1.8)	6 (2.0)	4 (1.4)	
Bleeding, *N* (%)	8 (1.6)	3 (1.0)	3 (1.1)	
Moderate bruising, *N* (%)	54 (10.7)	35 (11.9)	20 (7.0)	
Severe, *N* (%)	10 (2.0)	6 (2.0)	3 (1.1)	
Major complication, *N* (%)	2 (0.4)	1 (0.3)	1 (0.4)	0.419
Pseudoaneurysm, *N* (%)	1 (0.2)	1 (0.3)	1 (0.4)	
Arteriovenous fistula, *N* (%)	0 (0.0)	0 (0.0)	0 (0.0)	
Artery stenosis, *N* (%)	1 (0.2)	0 (0.0)	0 (0.0)	

SD, standard deviation.

In the patients without treatment of antiplatelet agents or anticoagulant, it showed no significant difference in mild bleeding, mild hematoma, and moderate/ severe subcutaneous bruising among three subgroups (A1, B1, and C1). And no major complications occurred in these three subgroups (A1, B1, and C1). In the patients who underwent treatment of antiplatelet agents or anticoagulant, the subgroup C2 showed a lower rate of minor and major complications than the other two subgroups (A2 and B2) (Table 3).

**Table 3:** Association between methods of hemostasis with complications in patients treated with antiplatelet agents and anticoagulant.

Patients treated with antiplatelet agents/anticoagulant	OR (95%CI)	*p* value
Methods of hemostasis		<0.001
Subgroup A2	Ref	
Subgroup B2	1.370 (0.862–2.178)	0.183
Subgroup C2	0.383 (0.226–0.652)	<0.001

In the cases with mild bleeding, mild hematoma and moderate/severe subcutaneous bruising, a prolonged placement of a sand bag was effective to control the complications, and no additional surgical treatment was required. In the three patients who developed pseudoaneurysms, ultrasound examination or CT angiography was performed to confirm the diagnosis. Then repair of the femoral artery was carried out in these patients. Severe femoral artery stenosis occurred in one patient. The patient complained progressive pain of right lower limb post operation and ischemic signs including decreased skin temperature and pulselessness were found in the right foot. Ultrasound examination was performed and showed severe stenosis of the right femoral artery. Emergency surgery was arranged to the patient and then the femoral artery was revascularized.

## Discussion

In this study, ECD was demonstrated to be feasible and safe for hemostasis of femoral arterial puncture site in patients undergoing percutaneous endovascular procedure. ECD could reduce bed rest time, increase the level of patient’s comfort, and result in less minor or major complications. Furthermore, the combined application of ECD and VCD could enhance the effect of hemostasis in patients treated with anticoagulant and antiplatelet agents.

Manual compression is the gold standard for hemostasis at the puncture site of the common femoral artery after percutaneous endovascular procedure. But it is laborious and time-consuming, and increases the level of patient’s discomfort, especially in the patients treated with anticoagulant and antiplatelet agents. To avoid these disadvantages of manual compression, some hemostatic methods including VCDs and mechanical compression devices have been designed. VCDs include collagen plug/sponge devices, suture-mediated devices, staple/clip devices, and patch/pad technology (3). Koreny et al. (11) in their systematic review showed that there were no significant differences in the relative risk of groin hematoma, bleeding, development of arteriovenous fistula, or development of pseudoaneurysm between any VCD and standard manual compression, but the time to hemostasis was shorter in the group with VCD. However, VCDs may increase the risk of femoral artery stenosis and affect a secondary puncture of the femoral artery (12). Also, neither the ProGlide nor the AngioSeal is suitable for patients with severe calcification at the femoral puncture site (13).

The Efinger ECD applied in the present study had the same basic principle of hemostasis as manual compression actually, but it was more time-saving and labor-saving for intervention physician than manual compression. In our practice, about 3–5 min were required to achieve initial hemostasis by exact application of ECD. And this device was relatively easy to use for hemostasis without a prolonged learning curve. Also, the device could provide greater stability in the inguinal area during bed rest compared with manual compression. Especially in emergent scenarios of acute cerebrovascular diseases, e.g., aneurysmal subarachnoid hemorrhage, emergent angiography and endovascular treatment are usually required (14). Considering the time-saving, easy to use, and stable characteristics of ECD, we thought application of ECD would also be a feasible choice of hemostasis in emergent scenarios.

The overall success rate of hemostasis in the Efinger ECD-treated group was 82.7%, which was comparable to that in the Proglide VCD-treated group. Based on the analysis of the association between the arterial sheath size and the success rate of hemostasis in the Efinger ECD-treated group, we found that the hemostatic effect of the device was not affected by the arterial sheath size. The rate of major and minor complications related to the application of Efinger ECD was 17.0% and 0.3%, respectively. There was no significant difference between the Efinger ECD-treated group and the Proglide VCD-treated group. Most complications in patients using the Efinger ECD were mild bleeding, small hematoma, or moderate subcutaneous bruising, which were managed conservatively. Compared with VCDs, the Efinger ECD could avoid the risk of femoral artery stenosis and embolism since there was no foreign body implantation related with ECD. Furthermore, the hemostatic effect of ECD was not affected by the local calcification of femoral artery. Additionally, Efinger ECD, which was about $ 65, costed less than Proglide VCD, indicating that application of the Efinger ECD could be a cost-effective hemostasis method.

Although blood loss from diagnostic angiography is marginal, hemoglobin decrease during endovascular interventions is not to be ignored (15). Pre-operative use of antiplatelet/anticoagulant appears to increase bleeding risk and may require closer patient monitoring (15). After further analyzing the level of hemoglobin decrease (> 10 g/L) and the rate of minor and major complications of different methods of hemostasis in each subgroup, we found that combined application of ProGlide VCD and Efinger ECD could achieve a better hemostatic effect than using ProGlide VCD or Efinger ECD alone in patients who underwent treatment of antiplatelet agents and anticoagulant. But the combined method of hemostasis had no advantage in patients without treatment of antiplatelet agents or anticoagulant, compared with other two subgroups (A1 and B1). Thus, considering the cost of Efinger ECD and the better hemostatic effect of combined hemostasis methods, we thought that it would be worth performing combined application of these two hemostasis devices in patients with antiplatelet agents and anticoagulant treatment. However, there was no need to apply combined method of hemostasis in patients without treatment of antiplatelet agents or anticoagulant. Also, these findings indicated that combined application of VCD and ECD was an effective and feasible hemostatic method with higher success rate of hemostasis in patients with prolonged TT, PT, and APTT. And speculatively, in acute ischemic stroke patients who would receive intravenous thrombolysis (alteplase/tenecteplase) and bridging mechanical thrombectomy, combined application of these two devices could also work well. But it needs to be confirmed in further studies.

This study has several limitations. First, the present study was retrospective, contrasting with the well-designed, prospective, randomized comparative clinical trials. Methodological bias could influence the possible advantage of one treatment in comparison with others. Second, Doppler ultrasonography and CT angiography were not performed in all the patients for evaluating complications associated with hemostasis. Therefore, complications such as pseudoaneurysm, arteriovenous fistula or arterial stenosis might be masked and under-evaluated. Also, the present study did not evaluate the late complications of the puncture site, which needed to be evaluated in further studies.

## Conclusion

The Efinger ECD is a safe and effective tool for hemostasis in femoral artery puncture site in percutaneous endovascular procedure with high clinical success and low complication rates. A combined application of VCD and ECD can achieve a better result of hemostasis with reduced immobilization time and increased level of patient’s comfort.

## Data Availability

The original contributions presented in the study are included in the article/supplementary material, further inquiries can be directed to the corresponding author.
